# Acetate-containing supernatants from industrial off-gas cultivation enabling high-value product formation with established and emerging production organisms

**DOI:** 10.1186/s13068-025-02732-4

**Published:** 2026-01-09

**Authors:** Lara Strehl, Paul Richter, Jathurshan Panchalingam, Robert Dinger, Franziska Höfele, Frank R. Bengelsdorf, Marcel Mann

**Affiliations:** 1 Aachener Verfahrenstechnik – Chair of Biochemical Engineering, RWTH Aachen University, 52074 Aachen, Germany; 2 Bioeconomy Science Center (BioSC), 52425 Jülich, Germany; 3 Institute of Molecular Biology and Biotechnology of Prokaryotes, Ulm University, Ulm, Germany

## Abstract

Gas fermentation offers a sustainable alternative for valorizing climate-active gases and industrial off-gases. Currently, these gases require energy-intensive purification steps before they can be used in chemical processes such as Fischer–Tropsch synthesis. In gas fermentation, anaerobic bacteria produce acetate from industrial off-gases. Compared to chemical processes, the anaerobic bacteria offer greater tolerance to varying gas concentrations and impurities. One major product of these anaerobic valorization processes is acetate, which can be used as a co-substrate in a variety of biological processes. This study evaluates *Corynebacterium glutamicum* and *Ustilago maydis* in benchtop cultivations using 10–20% (v/v) sterile-filtered acetate-rich supernatants from *Acetobacterium woodii* fermentation to produce L-lysine and triglycerides. Partial substitution of glucose with these supernatants supported robust growth and required no additional purification beyond sterile filtration. *C. glutamicum* achieved a L-lysine concentration of 3.5 ± 0.27 g∙L^−1^ and exhibited a diauxic growth pattern on glucose and acetate. In *U. maydis*, supernatant addition shortened the lag phase by approximately 2 h but reduced triglyceride yields modestly due to higher nitrogen availability. Optimizing the nitrogen-to-carbon ratio in benchtop fermentations resulted in a triglyceride concentration of 12.75 ± 1.17 g∙L^−1^, demonstrating the feasibility of this approach. Collectively, the results demonstrate a viable method for replacing a portion of refined glucose with acetate-rich supernatants, thereby enabling a cost-efficient integration of anaerobic gas valorization with aerobic biomanufacturing.

**Graphical Abstract:**

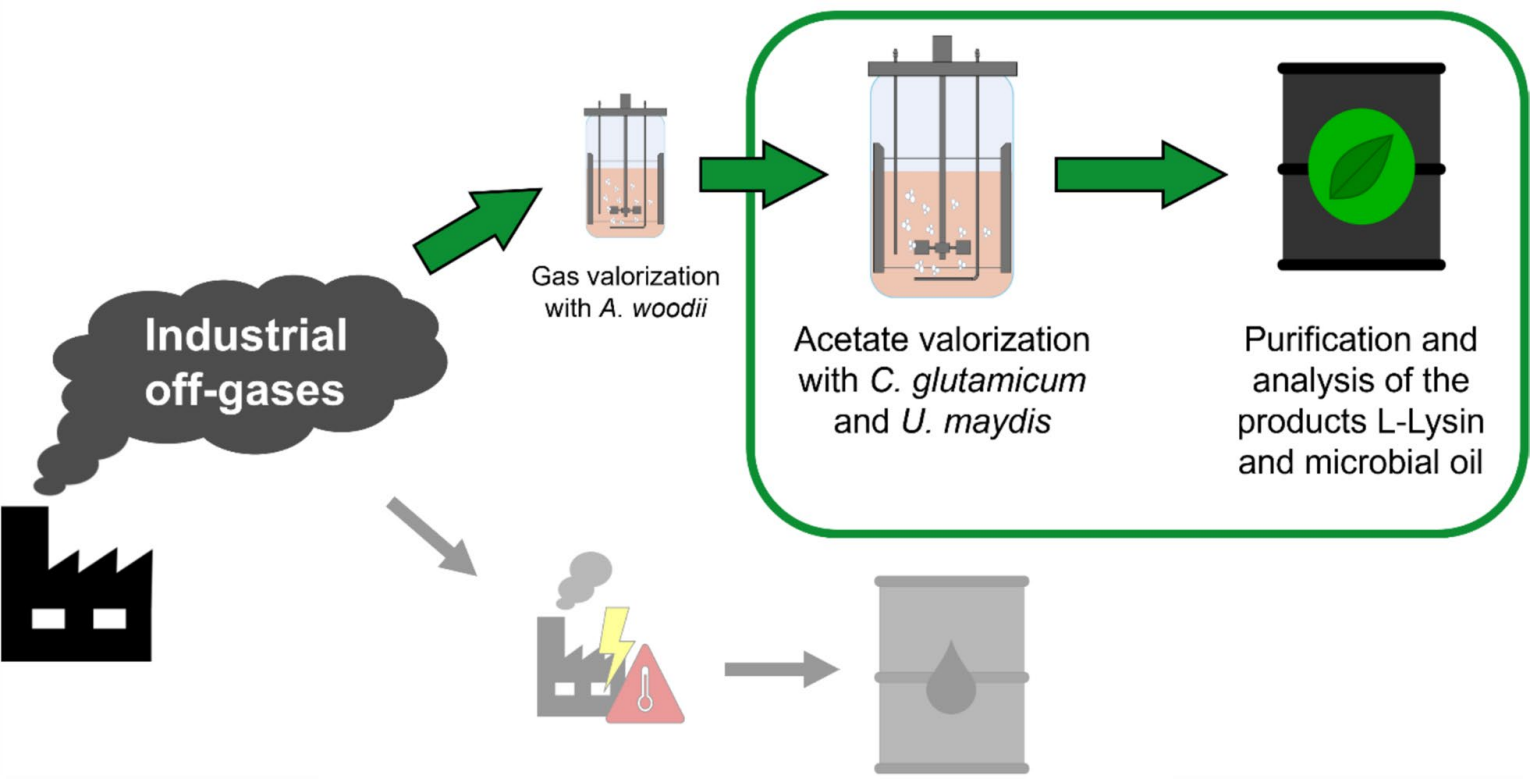

**Supplementary Information:**

The online version contains supplementary material available at 10.1186/s13068-025-02732-4.

## Introduction

Nowadays, an increasing number of chemical production processes are being examined for their environmental impact and high energy consumption. As sustainability becomes a key priority, industries are under pressure to develop clean and more efficient alternatives [1, 2]. Economic trade-offs and social demands not only drive the direct production of various goods but also emphasize the need to valorize by-products and waste streams for their production. One such waste stream is industrial off-gases. These gases are typically produced in energy-intensive industrial processes that are often irreplaceable for society, such as steel manufacturing, oil refining, or cement production [3]. Industrial off-gases are characterized by a high degree of variability in terms of their composition and quality, attributable to the influence of various process parameters. The gas composition, which predominantly consists of carbon monoxide (CO), hydrogen (H₂), and carbon dioxide (CO₂), exhibits significant variations depending on the specific source process. These variations also include the presence of impurities. Despite the various compositions, many industrial off-gases are already a key feedstock in several well-established chemical processes, such as the production of alcohols or fuel-grade hydrocarbons [4, 5]. However, high pressures and temperatures are required for chemical processing, making the entire process energy-intensive. Additionally, the catalysts used in these processes are specifically designed for precise H₂/CO ratios and high-purity feedstocks, adding further complexity to their utilization [6]. As the ratio in question cannot be guaranteed due to the inherent variability of the processes involved and the potential for impurities to occur, an additional intensive purification step is required. As a result, the chemical process is limited in its applicability and sustainability.

A valid alternative with comparatively low energy requirements, which can cope with some impurities and fluctuations in gas composition, is the biotechnological approach of gas fermentation [7]. Specific bacteria, such as acetogens, are capable of converting off-gases into a variety of products, including alcohols and acids [7, 8]. Acetic acid remains the most abundant product that can be produced by acetogens from different gas concentrations. It is a versatile intermediate that can serve as a precursor for a multitude of chemical and biological processes. For instance, acetic acid can be further converted into a variety of industrial chemicals through established chemical synthesis routes but can also be used directly as an additive, for example, in food products [9]. From a biological perspective, acetic acid can be metabolized by a vast array of microorganisms, resulting in the production of a range of bioproducts, including amino acids like L-lysine, triglycerides, and other bio-based products [7, 10–12].

The objective of this study is to investigate the potential of utilizing acetic acid directly derived from gas fermentation of *Acetobacterium woodii* as a substrate for two microbial production hosts, *Corynebacterium glutamicum* and *Ustilago maydis*. Such sequential fermentations would have the advantage of a significantly expanded product range, as well as potentially reduced media costs, if the medium can directly be used in an industrial setup. This utilization of the supernatants could then turn the C1 gases into a valuable product, such as amino acids or lipids.

*C. glutamicum* is known for its capability to produce amino acids, including glutamate and L-lysine, which are frequently used by the food and pharmaceutical industry [13]. In the context of this work, L-lysine is produced as an example product based on acetate as a secondary carbon source besides glucose. Normally, monosaccharides and disaccharides such as glucose or sucrose are used for their production. Second-generation feedstocks such as molasses or the acetate-containing supernatants, as outlined in this study, are rarely described in the current literature [14–17]. The basidiomycete *U. maydis*, a dimorphic fungus, can produce an array of bio-based chemicals, such as itaconic acid, glycolipids, and triglycerides, which find applications in many industries. In the context of this work, *U. maydis* is employed to produce triglycerides, using the acetate as substrate. For this purpose, the specific *U. maydis* strain MB215Δcyp1Δemt1 is employed, which was optimized for triglyceride production through two genetic knockouts. The triglyceride production from acetate-containing supernatants has already been shown for other yeasts [18], as for *U. maydis* utilizing pure acetate [11]. To our knowledge, direct use of acetate-containing off-gas supernatants without purification in *U. maydis* has not been reported. This positions acetate-rich off-gas supernatants as an immediately usable co-substrate stream for fungal lipid production. Expensive carbon sources such as glucose could be replaced by low-cost substrate acetate. The supernatants also contain micro and macronutrients that are not fully consumed by *A. woodii* in the previous fermentation. Consequently, the media costs in the sequential fermentation stage can be reduced, making the overall process more competitive. There is a high probability that acetate can be more readily incorporated into the fatty acid synthesis pathway of *U. maydis*, which could potentially yield an increased yield [11]. In this study, the acetate-containing supernatants were sterilized by filtration before subsequent fermentation to facilitate shipment and to maintain axenic conditions during laboratory cultivation. This measure served to standardize experimental handling and does not imply that identical sterilization would be required at an industrial scale, where media formulation and hygienic design would be optimized anyway.

## Material and methods

### Industrial off-gas valorization

The preliminary fermentation experiments investigating the metabolization of industrial off-gases with *A. woodii* are already published, with detailed data, including acetate concentrations by Höfele et al. in 2023. The experiments were conducted at the Institute of Molecular Biology and Biotechnology of Prokaryotes, Ulm University, Germany. The acetate-containing supernatants were sterilized by filtration prior to shipment and usage in the presented experiments. Additional information on the experimental setup is presented in the supporting information. The online and offline data of this preliminary gas fermentation can be found in Höfele & Dürre 2023 and in Figure S1 [19].

### Microorganism for the valorization of acetate-containing supernatants

The experiments to utilize acetate-containing supernatants from *A.* *woodii* fermentation from gas fermentation were conducted at the chair of Bioprocess Engineering at the RWTH Aachen University. For the assessment of a bacterial system, the strain *Corynebacterium glutamicum* DM 1933 was employed, which resulted from preliminary cloning and screening experiments conducted by Blombach et al. in 2009 [20]. As a fungal chassis organism, *Ustilago maydis* MB215Δcyp1Δemt1 [21] deposited at DSMZ as MB215cyp1emt1 (DSM17147) was used as a model organism. Throughout this work, the strains will be called *C. glutamicum* and *U. maydis*. The strains were stored in a 9 g∙L^−1^ sodium chloride solution with a glycerol concentration of 500 g∙L^−1^ at -80 °C.

### Media composition for L-lysine production with *C. glutamicum*

The *C. glutamicum* preculture was grown in YPG complex medium (10 g∙L^−1^ yeast extract, 10 g∙L^−1^ peptone, 20 g∙L^−1^ glucose, 2.5 g∙L^−1^ NaCl, and 0.25 g∙L^−1^ MgSO_4_ ∙ 7 H_2_O). The main cultivation of *C. glutamicum* was conducted in a modified CG-XII medium [13, 22]. The medium consisted of 20 g∙L^−1^ glucose, 1 g∙L^−1^ KH_2_PO_4_, and 2 g∙L^−1^ K_2_HPO_4_, 0.25 g∙L^−1^ MgSO_2_ ∙ 7 H_2_O, 10 g∙L^−1^ (NH_4_)_2_SO_4,_ 20 g∙L^−1^, 21 g∙L^−1^ MOPS buffer, 2 g∙L^−1^ CO(NH_2_)_2_, 0.01 g∙L^−1^ CaCl ∙ 2H_2_O, 0.0002 g∙L^−1^ biotin, 1 mL∙L^−1^ trace solution, and 0.03 g∙L^−1^ 3,4-dihydroxybenzoic acid. The trace element solution was prepared with 10 g∙L^−1^ FeSO_4_ ∙ 7H_2_O, 10 g∙L^−1^ MnSO_4_ ∙ H_2_O, 1 g∙L^−1^ ZnSO_4_ ∙ 7H_2_O, 0.02 g∙L^−1^ CuSO_4_, and 0.002 g∙L^−1^ NiCl_2_ ∙ 6H_2_O. The trace element solution was adjusted to pH 1 with H_2_SO_4_ and stored at 4 °C. The 3,4-dihydroxybenzoic acid and biotin solution were stored at -20 °C. All media components were diluted in demineralized (DI) water, except for the 3,4-dihydroxybenzoic acid and the biotin solution. The biotin was diluted in 50% (v/v) 2-propanol-DI water solution, while the 3,4-dihydroxybenzoic acid was diluted in 10% (w/v) solution of NaOH-DI water. The pH value of the MgSO_4_ ∙ 7 H_2_O, (NH_4_)_2_SO_4_, KH_2_PO_4_, and K_2_HPO_4_ was adjusted to 7.25 with 5 M NaOH solution. When sterile-filtered, acetate-containing supernatant was added, the glucose concentration was proportionally reduced to maintain an equal total carbon input across conditions. The previously mentioned solution, as well as the MOPS and urea solution, were prepared separately, autoclaved, and stored at room temperature. All other media components were sterile-filtered, by using a 0.2 µm cut-off cellulose acetate membrane filter (VWR International GmbH, Darmstadt, Germany). The initial pH of the medium was adjusted with 5 M NaOH solution to 7.25.

### Media composition for triglyceride production with *U. maydis*

For all shake-flask cultivations with *U. maydis*, a modified Verduyn mineral medium [23] was used, with the following composition: 20 g∙L^−1^ glucose, 0.32 g∙L^−1^ (NH_4_)_2_SO_4_, 0.5 g∙L^−1^ KH_2_PO_4_, 0.2 g∙L^−1^ MgSO_4_ ∙ 7H_2_O, 0.01 g∙L^−1^ FeCl_3_ ∙ 6H_2_O and 1 mL∙L^−1^ trace element solution, which contained: 15 g∙L^−1^ EDTA, 3 g∙L^−1^ FeSO_4_· 7H_2_O, 0.84 g∙L^−1^ MnCl_2_ · 2H_2_O, 4.5 g∙L^−1^ ZnSO_4_ · 7H_2_O, 0.3 g∙L^−1^ CuSO_4_ · 5H_2_O, 0.3 g∙L^−1^ CoCl_2_ · 6H_2_O, 0.4 g∙L^−1^ Na_2_MoO_4_ · 2H_2_O, 4.5 g∙L^−1^ CaCl_2_· 2H_2_O, 1 g∙L^−1^ H_3_BO_3_ and 0.1 g∙L^−1^ KI. The (NH_4_)_2_SO_4_, MgSO_4,_ and KH_2_PO_4_ solutions were autoclaved and stored at room temperature. All other media components were sterile-filtered by using a 0.2 µm cut-off cellulose acetate membrane filter (VWR International GmbH, Darmstadt, Germany). The initial pH of the medium was adjusted with 5 M NaOH solution to 6.5. Depending on the cultivation, parts of the glucose were replaced by acetate-containing supernatants from *A. woodii* fermentation. When sterile-filtered acetate-containing supernatant was added, the glucose concentration was proportionally reduced to maintain an equal total carbon input across conditions.

The cultivation in a benchtop fermenter with *U. maydis* was performed using the same modified Verduyn medium with adjusted glucose and nitrogen content: 100 g∙L^−1^ glucose, 4 g∙L^−1^ (NH_4_)_2_SO_4_, 0.5 g∙L^−1^ KH_2_PO_4_, 0.2 g∙L^−1^ MgSO_4_ ∙ 7H_2_O, 0.01 g∙L^−1^ FeCl_3_ ∙ 6H_2_O, and 1 mL∙L^−1^ trace element solution. The acetate feed was realized, using a 100 g∙L^−1^ acetic acid stock solution, which was sterilized by filtration using a 0.2 µm cut-off cellulose acetate membrane filter (VWR International GmbH, Darmstadt, Germany).

### Shake-flask cultivation

The cultivations in shake flask scale with *C. glutamicum* and *U. maydis* were performed by using the in-house-built Respiration Activity Monitoring System (RAMOS), with unbaffled 250-mL shake flasks. Detailed information about the setup and technology can be found in the supplementary information or in Anderlei et al*.* 2001 and 2004 [24, 25]. Commercial versions of the RAMOS device can be acquired from Kühner AG (Birsfelden, Switzerland) or HiTec Zang GmbH (Herzogenrath, Germany). Precultures of both organisms were inoculated from cryo-cultures to an optical density at 600 nm (OD_600_) of 0.1 and harvested at the exponential growth phase. After washing with 9 g∙L^−1^ sodium chloride solution, the main culture was inoculated with the prepared preculture to an initial OD_600_ of 0.1. The shaking frequency was set to 350 rpm, the shaking diameter to 50 mm, and the temperature to 30 °C. A filling volume of 10 mL for *C. glutamicum* and of 20 mL for *U. maydis* was used.

### Bioreactor cultivation

The two-liter scale fermentation was conducted in a 2-L New Brunswick™ BioFlo^®^/CelliGen^®^ benchtop bioreactor (Eppendorf SE, Hamburg, Germany). The temperature was maintained at 30 °C. The dissolved oxygen tension (DOT) was determined using a VisiFerm^™^ DO 225 pO2 sensor (Hamilton Bonaduz AG, Bonaduz, Switzerland), and the agitation speed was adjusted to maintain a DOT of 30%. The pH value was measured using an EasyFerm Plus K8 200 pH sensor (Hamilton Bonaduz AG, Bonaduz, Switzerland). The Rosemount X-Stream NGA 2000 exhaust gas analyzer (Emerson Electric Co., St. Louis, USA) was employed to determine the O_2_ and CO_2_ concentrations, used to calculate the oxygen transfer rate (OTR), carbon dioxide transfer rate (CTR), and respiratory quotient (RQ). The feed was realized using an Ismatec Reglo Analog ISM830 peristaltic pump (VWR International GmbH, Darmstadt, Germany) with a feed rate of 0.2 ml·min^−1^. The pH was maintained at 6.5 using 5 M NaOH. During the feed phase, the culture pH was maintained at 6.5 using an online single-loop PID controller acting on a dedicated base-addition line.

### Optical density and pH measurement

The OD_600_ was measured photometrically at 600 nm with a spectrophotometer (Genesys 202, Thermo Scientific, Darmstadt, Germany). Samples were diluted, if necessary, with 0.9%(w/v) NaCl solution.

The pH was measured with a pH meter (HI2211 pH/ORP meter, HANNA Instruments, Woonsocket, USA).

### HPLC analysis for glucose, acetate and L-lysine

The determination of glucose, acetate, and L-lysine was performed with high-performance liquid chromatography (HPLC). The measurement of glucose and acetate was conducted by utilizing a Shimadzu Prominence LC-20 HPLC system (Duisburg, Germany). An organic acid resin column (RezexROA-OrganicAcidH + (8%), 300 × 7.8 mm, Phenomenex Inc, USA) was applied in the HPLC for separation. The eluent used was 25 mM H_2_SO_4_ at a flow rate of 0.8 mL∙min^−1^ at 75 °C. The L-lysine concentration was measured in a HPLC equipped with a normal phase column with embedded acidic ion-pairing groups (Primesep S 4,6 × 150 mm, Sielc, USA). A flow rate of 0.8 mL∙min^−1^, a mobile phase with a 75:25 water-acetonitrile solution with 80 mM ammonium formate was used. The measurements were conducted at 50 °C with a Dionex Ultimate 3000 system (Thermo Scientific, USA). During the HPLC measurements, calibration curves were prepared using standards that covered the concentration range of 0 – 10 g∙L^−1^ of all analytes analyzed in this publication. Samples exceeding this range were appropriately diluted to ensure they fit the linear calibration interval. To maintain consistent measurement quality, standard solutions were analyzed at regular intervals between sample runs. Furthermore, the HPLC column was equilibrated with the respective eluent before each measurement to ensure stable chromatographic conditions.

### Triglyceride quantification

For the quantification of triglycerides, 2.4 mL of culture broth was sonicated using a Fisherbrand™ Model 120 Sonic Dismembrator equipped with a 1/8″ microtip (Fisher Scientific, Schwerte, Germany) to disrupt the cells. Subsequently, 2 mL of the crude extract was subjected to lipid extraction, as outlined by Matyash et al. (2008) [26], employing 5 mL of methyl tert-butyl ether (MTBE) and 1.5 mL of methanol. Following phase separation, the organic phase was transferred to pre-dried, pre-weighed glass vials and evaporated at room temperature. The vials were subsequently reweighed until mass consistency.

## Results and discussion

**Fig. 1:**
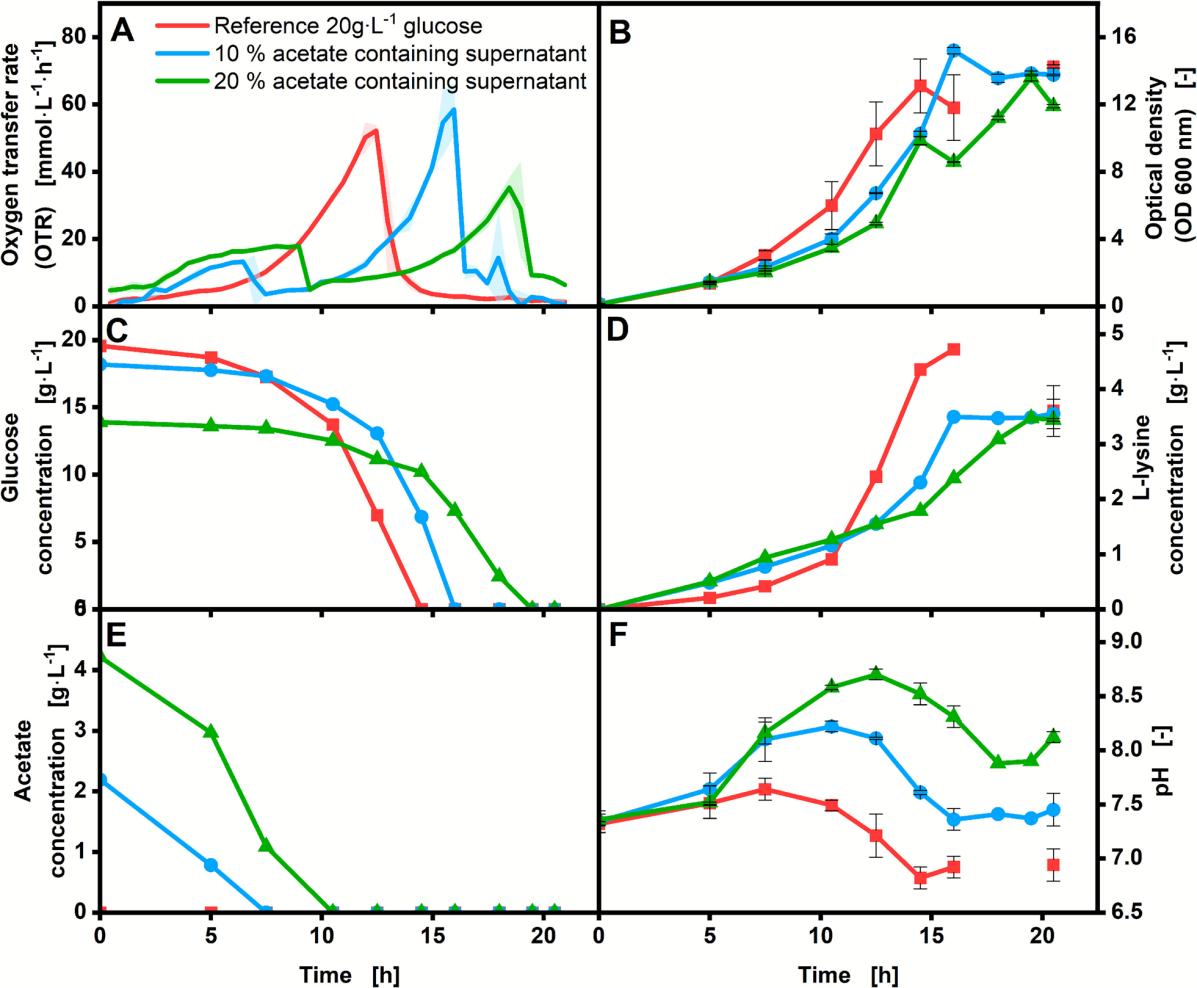
Production of L-lysine with *C. glutamicum* DM 1933 in CGXII medium with supplementation of acetate-containing supernatant. L-Lysine production with *C. glutamicum* DM 1933 reference (red), with 10% (blue) and 20% (green) acetate-containing supernatant supplementation in CG XII medium. Display of OTR (**A**), OD_600_ (**B**), glucose concentration (**C**), L-lysine concentration (**D**), acetate concentration (**E**), pH (**E**) over the cultivation time. Offline analysis (B + F) was performed with biological duplicates; error bars represent the min/max values of each measurement point. Final HPLC analysis (**C**–**E**) was performed in technical triplicate; error bars represent the standard deviation. Monitoring of the OTR was conducted with biological duplicates, with the average values represented as a line and the min/max values illustrated as error shadows. Cultivation conditions: 250-mL shake flask, V_L_ = 10 mL, d_0_ = 50 mm, n = 350 rpm, T = 30 °C, initial OD_600_ = 0.1, n = 2

**Fig. 2:**
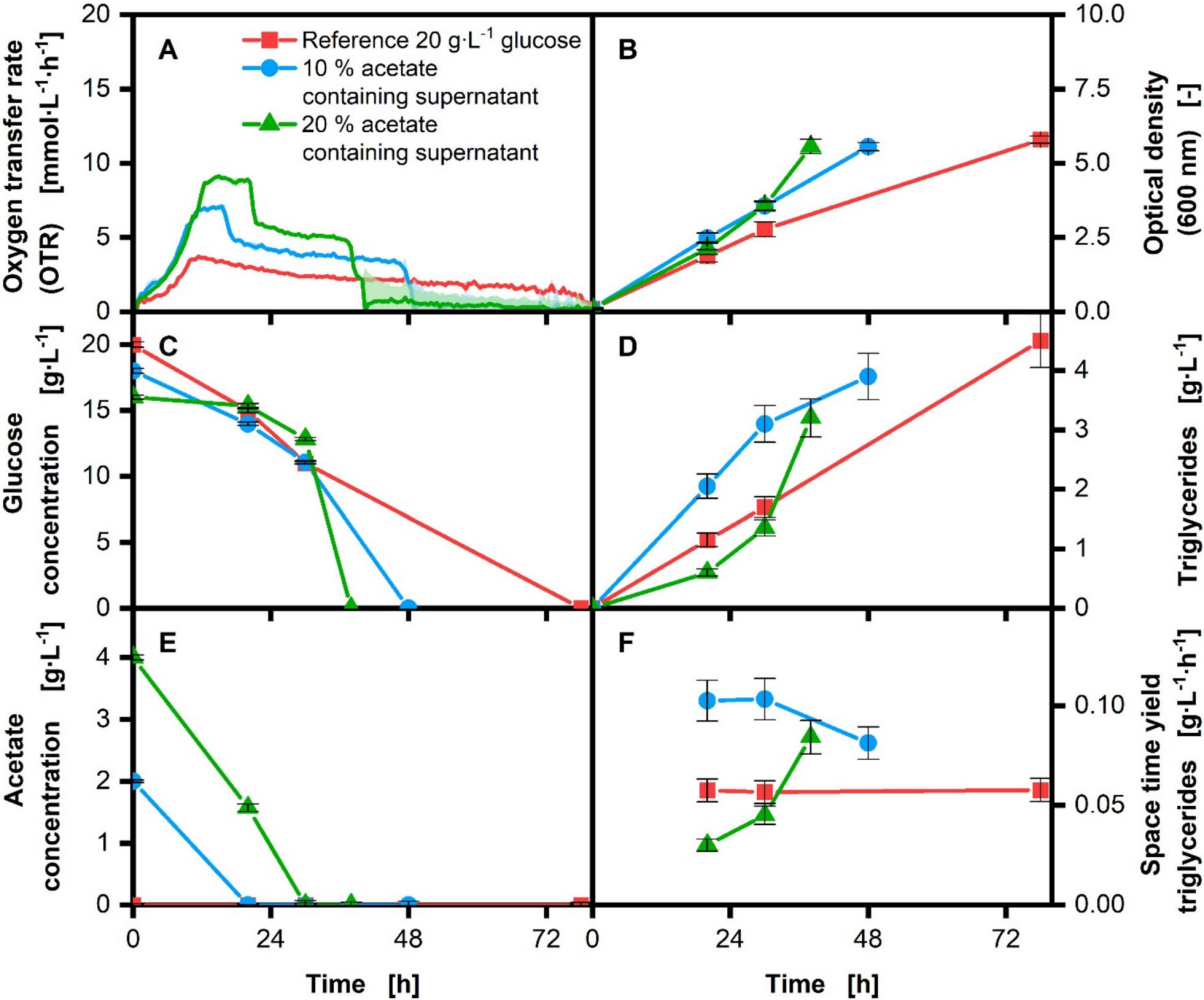
Shake-flask cultivation of *U. maydis* MB215Δcyp1Δemt1 with acetate supplementation for triglyceride production. Cultivation of *U. maydis* reference (red), with 10% (blue) and 20% (green) carbon-normalized acetate supplementation in modified Verduyn medium, realized by supernatant addition. Display of OTR (**A**), OD_600_ (**B**), glucose concentration (**C**), triglyceride concentration (**D**), acetate concentration (**E**), space time yield of triglyceride concentration (**F**) over the cultivation time. Offline analysis (B–E) was performed with biological duplicates and in technical duplicates; error bars represent the standard deviation. Monitoring of the OTR was conducted with biological duplicates, with the average values represented as a line and the min/max values illustrated as error shadows. The fourth offline sample was taken after the OTR (A) of the respective cultivation showed a steep decrease indicating carbon source depletion. Cultivation conditions: 250 mL shake flask, V_L_ = 20 mL, d0 = 50 mm, n = 350 rpm, T = 30 °C, initial optical density OD_600_ = 0.1, n = 2

The results of the experiments conducted with *C. glutamicum* and *U. maydis* are presented in Figures 1 and 2, respectively. This study aimed to evaluate whether similar product titers could be achieved by substituting different volumes of glucose with acetate-containing supernatant derived from the gas fermentation of *A.* *woodii*. Specifically, 10% and 20% of the glucose in the respective media were replaced with acetate from the supernatant (further referred to as 10% and 20% supplemented supernatant). A control experiment utilizing glucose as the sole carbon source was also conducted for comparison. The study focused on characterizing the production of L-lysine with *C. glutamicum* and triglyceride production in *U. maydis*. In addition to the product concentrations, the online and offline parameters, OTR, optical density (OD), glucose and acetate concentrations, and the pH were analyzed for the process characterization.

### L-Lysine production with *C. glutamicum*

In Fig. 1 the results of the cultivation with *C. glutamicum* of CG XII medium without supplementation of acetate-containing supernatant (reference) (red) and with a supplementation of 10% (blue) and 20% (green) acetate-containing supernatant are displayed.

The OTR curves (Fig. 1A) for cultures supplemented with 10% (blue) and 20% (green) acetate-containing supernatant exhibit distinct peaks at 6.5 h and 9 h, respectively, within the first 10 h of cultivation, reflecting acetate metabolism. This observation was further confirmed by HPLC measurements (Fig. 1C, D, E). However, the OTR profiles do not display a sharp peak; instead, they exhibit a plateau for both cultivations, which indicates a limitation or inhibition [25]. The increase in pH could cause a pH inhibition, as *C. glutamicum* is a neutralophilic bacterium tolerating a maximum pH value of around 9 [25, 27]. Compared with the measured pH in Fig. 1 (F), the maximal measured pH of 8.7 of the cultivation with 20% supplemented acetate-containing supernatant is close to the upper limit of the tolerance range, suggesting that this already inhibits metabolism and respiration. The second OTR peaks for cultures supplemented with 10% and 20% acetate-containing supernatant occur at 16 h and 18.5 h, respectively, and can be attributed to glucose consumption. The glucose peak of cultivations with 20% supplemented acetate-containing supernatant is lower due to metabolic effects caused by the pH. The HPLC data show that acetate and glucose are metabolized one after the other, with acetate being metabolized first (Fig. 1C, E). The OTR and HPLC data show a diauxic growth of *C. glutamicum* on acetate and glucose, where acetate is the preferred substrate, according to the growth rate (Figure S2) [28]. Previous studies have shown that the OTR is a reliable indicator of diauxic growth in microorganisms [29, 30]. In this study, the occurrence of diauxic growth is further confirmed by complementary HPLC measurements. Even though Wendisch et al. could see in their work a non-diauxic growth on acetate–glucose mixtures for *C. glutamicum* ATCC 13032 WT [31]. This disparity could be due to the different strains and cultivation conditions used in their work. The diauxic growth in *C.* *glutamicum* can be explained by its metabolic pathways. Acetate can enter the cell either via passive diffusion or through active transport [32], after which it is enzymatically converted to acetyl-CoA and subsequently metabolized into isocitrate via the TCA cycle [33]. Acetate metabolism is primarily regulated by the transcriptional regulators RamA and RamB. RamA is activated when acetate serves as the carbon source, whereas RamB functions as a negative transcriptional regulator of RamA-associated genes in the presence of glucose [33–36]. This regulatory scheme would generally argue against diauxic growth. However, in the strain used here, *C. glutamicum* DM1933, relevant gene sequences within this metabolic pathway are altered [20], which could account for the observed diauxic growth on acetate and glucose. Additionally, fewer metabolic steps are required to channel acetate into the TCA cycle, which may explain why acetate was metabolized first under these conditions. Also, diauxic growth for *C. glutamicum* could previously be seen at glucose and glutamate as carbon sources [37, 38]. Through the pH inhibition and the metabolic switch during the diauxic growth, a prolonged process time was present. At the cultivation with glucose as the sole carbon source (reference, 20 g·L^−1^ glucose, red), only one peak in the OTR was measured at 12.5 h, showing the consumption of glucose. During the first 12.5 h of the cultivation, the pH values increase for all approaches but to varying degrees (Fig. 1F). With higher amounts of initial acetate concentration (10% and 20% acetate-containing supernatant), the higher the pH peak. The increase in pH, already during the reference cultivation, can be attributed to the release of ammonium ions by hydrolysis of urea [39]. The stronger increase in pH at 10% and 20% supplemented supernatant is a result of the consumption of acetate. A decrease in pH was observed in all experimental approaches: for the reference after 7.5 h, for the 10% supplemented supernatant after 10.5 h, and for the 20% supplemented supernatant after 12.5 h. This phenomenon can be attributed to the formation of organic acids [40]. A similar biomass concentration for all three approaches could be achieved at the end of the fermentation (Fig. 1B). For 10% and 20% supplemented supernatant, the L-lysine concentration was similar (3.49 and 3.47 g∙L^−1^). Compared to the reference cultivation, a L-lysine concentration of 4.35 g∙L^−1^ was measured (Fig. 1D), showing a higher product formation, as well as a higher space time yield (STY) (Figure S3). The space time yield of the cultivations was calculated for every time point of sampling by dividing the delta of the product (L-lysine or triglyceride) concentration (Δc_product_) by the delta of production time (Δt) (Eq. 1):

1 $${\mathrm{STY}}_{product} =\frac{{\Delta c}_{product}}{\Delta t}$$

This could be related to less optimal pH during the cultivations with 10% and 20% supplemented supernatant. However, a higher product yield was demonstrated during the metabolization of acetate, compared to glucose (Figure S4). Further studies could investigate the potential for pH regulation to achieve the same L-lysine concentration while maintaining a pH range comparable to that observed in the reference cultivation. This study, nonetheless, demonstrated that L-lysine can be produced from the acetate-containing supernatant derived from gas fermentation at concentrations comparable to those obtained from cultivations utilizing glucose as the sole carbon source.

### Triglyceride production with *U. maydis*

As another production system of interest, we investigated the triglyceride production of the basidiomycete *U. maydis*. Based on preliminary growth tests (see supplementary information Figure S5), a maximal of 20% supernatant concentration was selected to remain within a range where no inhibitory effects on *U. maydis* growth occur. The results of supplementation of the acetate-containing supernatants are presented in Fig. 2.

In contrast to *C. glutamicum*, *U. maydis* shows a different behavior in the OTR courses (A). For the reference cultivation, it can be observed that the oxygen demand of the culture exhibits an exponential increase, reaching its highest level after approximately 12 h. At this time point, the nitrogen in the medium is depleted and the triglyceride production starts [41, 42]. Following this metabolic shift, the OTR slowly decreases until the carbon source is fully depleted. This can be seen in the glucose concentration (C), with a slow and steady decrease after the growth phase, during which it was consumed faster. This steady consumption can also be observed in the space time yield (STY) of product triglycerides. The STY was calculated by dividing the triglycerides concentration gathered from the gravimetric measurement by the timepoint of the respective sample. After the growth phase, there is a significant increase to an STY of 0.05 g∙L^−1^∙h^−1^ followed by consistent oil production with the same yield until the end of cultivation.

A comparison with the cultivations with supernatant supplementation reveals similar exponential increases in respiratory activity in the beginning of cultivation, yet different maximum OTR values. The reference cultivation exhibited the lowest OTR peak at 3.7 mmol∙L^−1^∙h^−1^, followed by 10 vol% and 20 vol% supplemented supernatant. The elevated OTR values observed with higher supernatant volume can be attributed to the presence of small amounts of nitrogen in the supplemented supernatants. This can be explained as in the used minimal‐salts reference medium, nitrogen is the sole limiting nutrient. The additional nitrogen from the supernatants then results in higher biomass formation and oxygen consumption. This OTR phenomenon is well established and has been widely documented in literature [43, 44]. However, regarding the growth phase, no negative effects of the supernatant addition can be observed. All cultivations show growth rates ranging from 0.20—0.22 h^−1^ which are common for the given cultivation conditions [41]. Furthermore, it is noteworthy that no diauxic behavior could be observed in the OTR, which allows for the assumption that acetate and glucose can be metabolized in parallel.

In the presented cultivations, the nitrogen availability in the medium is limited. When more supernatant is added, nitrogen limitation occurs later in the process. This delay occurs due to the presence of small amounts of nitrogen source in the supernatants. While delayed nitrogen limitation may have adverse implications for the presented triglyceride production processes, it could be addressed beforehand by appropriately reducing nitrogen levels in the experimental design. However, higher nitrogen availability should result in accelerated and enhanced biomass formation (B) and up to a certain amount in increased space time yield of produced triglyceride (F) (see supplementary information Figure S6 and S7).

Although the final OD measurement values are comparable, intracellularly formed triglycerides influence the optical volume of the cells and therefore also the OD measurement [45]. This results in a significantly higher OD being measured, particularly in later cultivation stages. Furthermore, despite nitrogen limitation, the OD continues to increase, providing additional evidence in support of this hypothesis.

Acetate (E) is consumed before or in parallel to glucose, leading to minimal glucose consumption during the 20 vol% supernatant supplementation throughout the growth phase (C). This potential parallel consumption was already seen in *U. maydis* [29] and could be beneficial for a fed-batch process, as it does not inhibit the uptake of other carbon sources, thus enhancing overall metabolic flux towards the product.

A comparison of the triglyceride concentration shows that with 10% and 20% acetate supplementation, triglyceride production is faster due to increased biomass. However, the triglyceride concentration (D) at the end of the experiment is lower, because more of the carbon source is diverted towards biomass growth rather than triglyceride accumulation. Accordingly, the reference production process is slower but reaches a higher absolute triglyceride concentration, due to a carbon-to-nitrogen allocation which is more focused on triglyceride production than growth. However, when setting up a fermentation process, time savings must be considered, as an increase in cultivation time could reduce the process’s efficiency. Therefore, the space time yield of the cultivations was calculated for every time point of sampling using Eq. 1.

In the reference cultivation, a constant space–time yield of around 0.057 g∙L^−1^∙h^−1^ could be observed during the whole cultivation. With an increasing supplementation volume and thus also with an increasing nitrogen concentration, the space–time yield reacts. Thus, the 10% supplementation shows a higher yield of 0.10 g∙L^−1^∙h^−1^ during the first two sampling points and a decreasing yield towards the end of the cultivation. The 20% supplementation shows a steadily increasing yield, which starts at 0.03 g∙L^−1^∙h^−1^ and increases until the end of cultivation to 0.08 g∙L^−1^∙h^−1^. The slow product formation at the beginning might be caused by longer biomass formation due to the higher nitrogen concentration introduced by adding the supernatant. Generally, it could be shown that the presence of acetate-containing supernatants nearly doubles the space–time yield of the triglyceride production process, although too much supernatant associated nitrogen can reduce the space–time yield.

These results underscore the importance of optimizing nitrogen and carbon sources in the fermentation process to balance growth and product formation effectively. By carefully adjusting these parameters, it is possible to enhance overall triglyceride production efficiency, while using the acetate-containing supernatants. Given the difficulties that would be encountered in conducting a fed-batch fermentation with the supernatants containing acetate and a nitrogen source as a feed, the next section of the study involved replacing the supernatants with pure acetate in the form of acetic acid.

### Triglyceride production on pure acetate feed

**Fig. 3:**
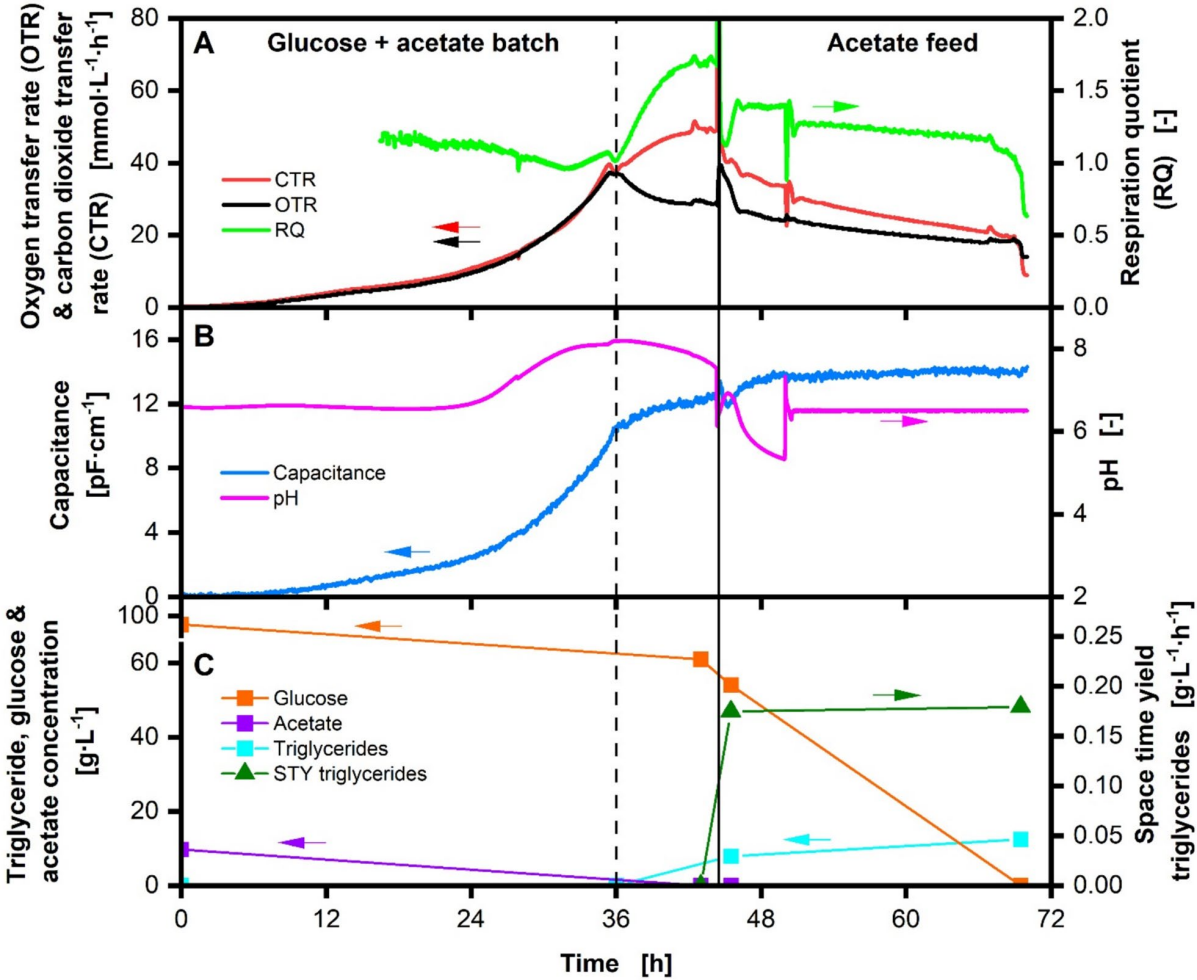
2-L benchtop cultivation of *U. maydis* MB215Δcyp1Δemt1 to assess the triglyceride production supported by an acetate fed batch. Displayed are the respiratory activity (OTR, CTR and RQ) (**A**), the capacitance and pH (**B**), and the educt and product concentrations (**C**) over the cultivation time. The dashed vertical lines represent depletion of nitrogen (visible in respiration activity (**A**) and capacitance (**B**)). The solid vertical line represents the start of the acetic acid feed. Offline analysis (**C**) was performed in technical triplicates; error bars represent the standard deviation of each measurement. Cultivation conditions: modified Verduyn medium; 2 L New Brunswick fermenter; V_L_ = 1.2 L, T = 30 °C, initial OD_600_ = 0.1; glucose concentration = 90 g·L^−1^; initial acetate concentration = 10 g·L^−1^; acetic acid feed concentration = 100 g·L^−1^; feed rate = 0.2 ml·min^−1^, n = 1

To test whether triglyceride production efficiency in *U. maydis* was increased in the shake flask experiment by the added acetate or other supernatant components, a bioreactor cultivation was performed with the addition of pure acetate. The cultivation was divided into a batch and a feeding phase to assure acetate abundance during triglyceride production. In the batch phase, 10% (10 g∙L^−1^) of the total carbon source was exchanged with acetate, as this ratio seemed the most promising in the first experiments. Shortly after the initial acetate was consumed, an acetate feed was started to ensure a sufficient supply during the triglyceride production phase. Online and offline data are presented in Fig. 3.

In comparison to the shake flask experiments, a prolonged lag phase is observable in both the OTR and the carbon dioxide transfer rate (CTR), with a duration of approximately 12 h (Fig. 3 A). This extension of the lag phase is likely due to the higher concentration of acetate used, which leads to a lower overall growth rate of 0.10 h^−1^, in comparison to 0.21 h^−1^ with reference conditions, with the same ratio but lower concentration [41]. Although some studies suggest lower acetate concentration for the growth of *U. maydis*, slow but efficient growth could be observed at this high acetate concentration of 10 g·L^−1^ [11, 46, 47]. Consequently, a slightly lower starting concentration of acetate may be selected in future fermentations, and the acetic acid feed should be started earlier. Nitrogen limitation becomes apparent at around 36 h, marked by a noticeable change in the OTR and by the vertical dashed lines. The respiratory quotient (RQ) visible in Fig. 3 A exceeds one shortly after the nitrogen source is depleted, indicating triglyceride production by the culture. Acetate consumption began during the growth phase, likely occurring alongside glucose utilization. This is suggested by the rising pH, which resulted from only basic pH control applied during the batch phase of the experiment. Capacitance measurement was employed to monitor biomass during fermentation, as it is not influenced by intracellular product accumulation when the scan frequency is kept constant, unlike cell dry weight or OD [48]. Capacitance measurement tracks the electrical properties of cells and provides real-time data on cell concentration and viability by detecting changes in the dielectric properties of the culture [49]. Capacitance measurements offer the advantage of clearly distinguishing the growth phase from the production phase. Therefore, this transition is not only reflected in the respiratory activity but also in a decline in biomass formation, as indicated by a change in the slope of the capacitance course [48].

The data obtained from HPLC verify the findings of the online measurements and demonstrate a simultaneous reduction in acetate and glucose levels during the growth phase. This indicates a parallel consumption of both carbon sources rather than a sequential utilization, which aligns with the absence of acetate accumulation during the feeding phase. Consequently, this approach offers a more precise understanding of acetate metabolism than the shake flask experiments. In shake flask cultivations, acetate was depleted early and therefore did not contribute significantly to triglyceride production. In contrast, the introduction of acetic acid as a feed in the bioreactor ensured that acetate concentrations remained above zero throughout the entire cultivation, allowing for its potential contribution during the production phase. This parallel acetate consumption may allow more efficient triglyceride production via the fed acetic acid. In this case, under nitrogen limitation in *U. maydis*, carbon routing toward acetyl-CoA occurs via two entry routes. Glucose is metabolized through glycolysis to pyruvate, which undergoes oxidative decarboxylation in the mitochondrial matrix to yield acetyl-CoA, which is the main precursor for fatty acid synthesis. Simultaneously, acetate moves across the plasma membrane by simple diffusion [50]. Intracellular acetate is then converted directly to acetyl-CoA, thus bypassing both glycolysis and pyruvate oxidation. Because acetate is a direct precursor of acetyl-CoA required for fatty acid synthesis, it is hypothesized that lipid synthesis would be favored and that triglyceride accumulation may proceed faster from acetate [11]. In this co-feeding setup, acetate thus supplies acetyl-CoA with fewer biochemical steps than glucose, enabling efficient channeling of carbon into fatty acid formation and subsequent triglyceride production under nitrogen-limited conditions.

After approximately 15 h of production or 51 h of cultivation, the triglyceride concentration reaches about 10 g∙L^−1^ (Fig. 3 C). This suggests that the sustained availability of acetate through the feed phase plays a role in supporting triglyceride synthesis under nitrogen-limited conditions.

This resulted in a rapid triglyceride production with acetate, starting from 0 g∙L^−1^ at the time of nitrogen depletion to 12.45 g∙L^−1^ at the end of the experiment. The resulting maximal STY of 0.17 g∙L^−1^∙h^−1^ is significantly higher than all STY of the shake flask experiments, with the highest STY at 0.1 g∙L^−1^∙h^−1^ (see Fig. 2 F). However, due to the high glucose concentration and the additional carbon source from the feed, the carbon yield of the produced triglycerides of 0.11 g∙g^−1^ is lower than the yields of comparable reference cultivations (0.2 g∙g^−1^) [41]. Optimization of the nitrogen content could potentially further enhance the STY and enable the triglyceride production in fed-batch mode, also when using acetate-containing supernatants. This experiment proved that triglyceride production with acetate co-feeding is possible and shows a comparatively good space–time yield.

## Conclusion

In this study, the ability of *C. glutamicum* DM 1933 and *U. maydis* MB215Δcyp1Δemt1 to utilize acetate-containing supernatants from gas fermentation was successfully demonstrated. *Corynebacterium glutamicum* exhibited diauxic growth when cultivated on a mixed carbon source of glucose and acetate, with no significant impact on final biomass concentration. L-Lysine production was detected under all tested conditions; however, a slight reduction in titer was observed in cultures with acetate-containing supernatants, likely due to pH shifts. This inhibitory effect may be mitigated by implementing pH control strategies. Notably, the product yield was enhanced in the presence of acetate. *U. maydis* showed improved biomass yield and shorter lag phases compared to glucose-only cultivation. Additionally, a STY of 0.1 g∙L^−1^∙h^−1^ was achieved, which was twice the STY in glucose-only cultivation. Adjusting the nitrogen-to-carbon ratio in future experiments could potentially enhance triglyceride production and STY even more.

This potential for acetate co-metabolization and higher STYs was further validated in a feeding experiment with *U. maydis*, where the initial addition of pure acetic acid led to a growth inhibition but also resulted in faster triglyceride production in a later stage. The STY could further be enhanced to a value of 0.17 g∙L^−1^∙h^−1^. This suggests that both acetate-containing supernatants and pure acetic acid could serve as valuable carbon sources.

Relative to studies employing pure acetate or pre-treated hydrolysates, our results show that as-received off-gas supernatants can sustain growth and product formation under carbon-normalized conditions and be co-consumed with glucose but also can modulate OTR partitioning via trace nitrogen carryover. In summary, this study demonstrates a sustainable approach to valorizing off-gas-derived acetate without extensive purification, offering an alternative to energy-intensive chemical synthesis. The integration of microbial fermentation with off-gas-derived feedstocks presents a viable method for producing valuable biochemicals while reducing reliance on fossil-based resources.

## Supplementary Information

Supplementary file 1.

## Data Availability

The datasets used and/or analyzed during the current study are available from the corresponding author on reasonable request.
