# Supplementary material for: Acetate-containing supernatants from industrial off-gas cultivation enabling high-value product formation with established and emerging production organisms
Source: Biotechnol Biofuels Bioprod. 2026 Jan 9;19:13. doi: 10.1186/s13068-025-02732-4 (PMC12859875; doi:10.1186/s13068-025-02732-4)
Supplement: Supplementary file 1 [file 13068_2025_2732_MOESM1_ESM.pdf]

<u>**Supporting information**</u>

Acetate-containing supernatants from industrial off-gas cultivation enabling high-value product formation with established and emerging production organisms

Lara Strehl[a, †], Paul Richter[a, b, †], Jathurshan Panchalingam[a], Robert Dinger[a], Franziska Höfele[c], Frank R. Bengelsdorf[c], Marcel Mann[a, b, *]

[a] Aachener Verfahrenstechnik – Chair of Biochemical Engineering, RWTH Aachen University, 52074 Aachen, Germany

[b] Bioeconomy Science Center (BioSC), 52425 Jülich, Germany

[c] Institute of Molecular Biology and Biotechnology of Prokaryotes, Ulm University, Ulm, Germany

[†] The authors contributed equally to this work

[*] Corresponding author, Aachener Verfahrenstechnik – Chair of Biochemical Engineering, RWTH Aachen University, 52074 Aachen, Germany, Email: marcel.mann@avt.rwth-aachen.de

Off-gas valorization experiments

Microorganism and media of the preliminary gas fermentation carried out at the Institute of Molecular Biology and Biotechnology of Prokaryotes at Ulm University in Germany. Corresponding cultivation data can be found in Höfele & Dürre (2023) [19].

The preceding gas fermentation was carried out using *Acetobacterium woodii* DSM 1030. Cultivation of the respective strain was performed anaerobically at 30 °C in modified DSM 135 medium consisting of 0.2 $g \cdot L^{-1}$ NH_4Cl, 1.76 $g \cdot L^{-1}$ KH_2PO_4, 8.44 $g \cdot L^{-1}$ K_2HPO_4, 1 $g \cdot L^{-1}$ yeast extract, 10 $g \cdot L^{-1}$ $NaHCO_3$, 0.3 $g \cdot L^{-1}$ HCl-cysteine · H_2O, 0.3 $g \cdot L^{-1}$ $Na_2S \cdot 9\ H_2O$, 2 $mL \cdot L^{-1}$ trace element solution, 2 $mL \cdot L^{-1}$ vitamin solution, and 1 $mg \cdot L^{-1}$ resazurin. The trace element solution was prepared by dissolving 12.8 $g \cdot L^{-1}$ nitrilotriacetate, 0.5 $g \cdot L^{-1}$ NaOH, 0.1 $g \cdot L^{-1}$ $MnCl_2 \cdot 4\ H_2O$, 5 $g \cdot L^{-1}$ NaCl, 2 $g \cdot L^{-1}$ $FeCl_2 \cdot 4\ H_2O$, 0.2 $g \cdot L^{-1}$ $CoCl_2 \cdot 6\ H_2O$, 70 $mg \cdot L^{-1}$ $ZnCl_2$, 2 $mg \cdot L^{-1}$ $CuCl_2 \cdot 2\ H_2O$, 6 $mg \cdot L^{-1}$ H_3BO_3, 36 $mg \cdot L^{-1}$ $Na_2MoO_4 \cdot 2\ H_2O$, 24 $mg \cdot L^{-1}$ $NiCl_2 \cdot 6\ H_2O$, 3 $mg \cdot L^{-1}$ $Na_2SeO_3 \cdot 5\ H_2O$, and 4 $mg \cdot L^{-1}$ $Na_2WO_4 \cdot 2\ H_2O$ in demineralized water. Vitamin solution consisted of 50 $mg \cdot L^{-1}$ HCl-pyridoxine, 50 $mg \cdot L^{-1}$ HCl-thiamine · H_2O, 50 $mg \cdot L^{-1}$ riboflavin, 50 $mg \cdot L^{-1}$ D-Ca-pantothenate, 25 $mg \cdot L^{-1}$ lipoic acid, 50 $mg \cdot L^{-1}$ p-aminobenzoate, 50 $mg \cdot L^{-1}$ nicotinic acid, 25 $mg \cdot L^{-1}$ vitamin B_{12}, 25 $mg \cdot L^{-1}$ biotin, and 25 $mg \cdot L^{-1}$ folic acid (Hoffmeister et al., 2016). After autoclaving the medium at 121 °C and 1.2 bar for 15 min, a carbon source and 1.3 mM $MgSO_4$ were added (Hoffmeister et al., 2016).

To cultivate *A. woodii* on the industrial off-gas mixture, 200 mL modified DSM 135 medium in 1000 mL serum bottles (SGD Pharma, Paris, France) was used. The gas phase of the serum bottles was changed to 1 bar overpressure CO_2 + H_2 (33 % + 67 %, MTI Industriegase AG, Elchingen, Germany). Cultures were transferred twice into fresh medium with CO_2 + H_2 in the headspace to adapt the metabolism of the respective strain to gaseous C_1 carbon sources. Autotrophic cultivation was performed anaerobically at 30 °C with shaking at 130 rpm. During growth the gas phase was refilled at a minimum pressure of 0.3 bar (overpressure).

Explanation of the RAMOS technology

The Respiration Activity MOnitoring System (RAMOS) enables the measurements of the respiratory activity in form of the oxygen transfer rate (OTR) in shake flasks. RAMOS flasks are connected to an external gas supply with sterile inlet/outlet filters and to an oxygen sensor. The system runs cyclically

through (i) a measuring phase, during which inlet and outlet valves are closed and the culture continues to breath, and (ii) a rinsing phase, during which the headspace is flushed with a defined gas mixture (air for the presented cultivations). The oxygen partial pressure in the sealed headspace decreases during the measuring phase solely due to biological oxygen uptake. The device computes the OTR from the slope of this decrease. The flask geometry and hydrodynamics are equivalent to standard unbaffled shake flasks; the lower (liquid) section is unmodified, the sensor does not contact the liquid, and the gassing reproduces the headspace composition of a cotton-plugged reference flask to ensure equivalence of culture conditions. Up to 8 flasks can be operated in parallel with automatic sensor-drift compensation.

$$OTR = \frac{\Delta p_{O_2}}{\Delta t} \cdot \frac{V_g}{R \cdot T \cdot V_l} \left[\frac{mol}{L \cdot h}\right] \qquad \text{(Equation S1)}$$

RAMOS determines OTR by a gas-phase mass balance during the sealed measuring interval. OTR per liquid volume is obtained from the time derivative of the headspace oxygen partial pressure (see Equation S1), corrected for (a) the compressible gas volume via the ideal gas law (ideal gas constant R) and (b) the dissolved-phase oxygen capacity via Henry's law at the operating temperature. Required parameters are the headspace volume V_g, liquid volume V_L, temperature T, and the difference of oxygen partial pressure) (Δp_{O2}) over time difference(Δt). The detailed construction and explanation of the measuring principle were published in 2001 by Anderlei et al..

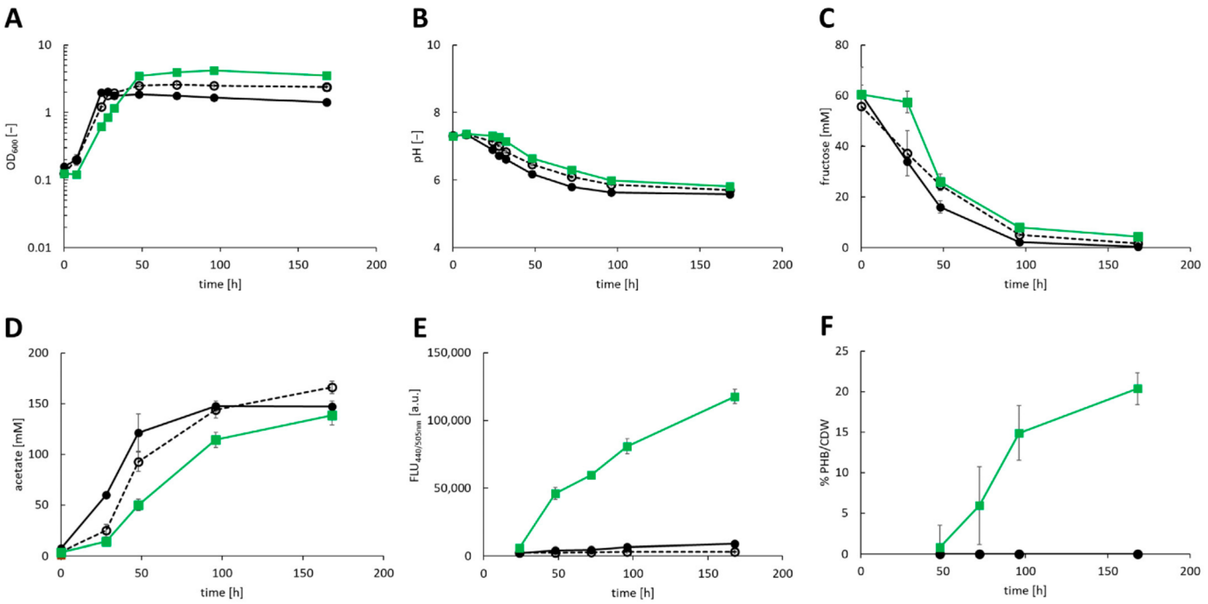

Figure S 1: **Results of *Acetobacterium woodii* cultivations over time**. Green: *A. woodii* DSM 1030; black: *A. woodii* [p83] strain. (A) Optical density at 600 nm (OD_{600}), (B) pH, (C) fructose consumption, (D) acetate production, (E) fluorescence intensity measured with LipidGreen2 assay, and (F) PHB production. The data represent growth in triplicate. Figure was taken from [19]. The supernatant from the cultivation of *A. woodii* DSM 1030 was used.

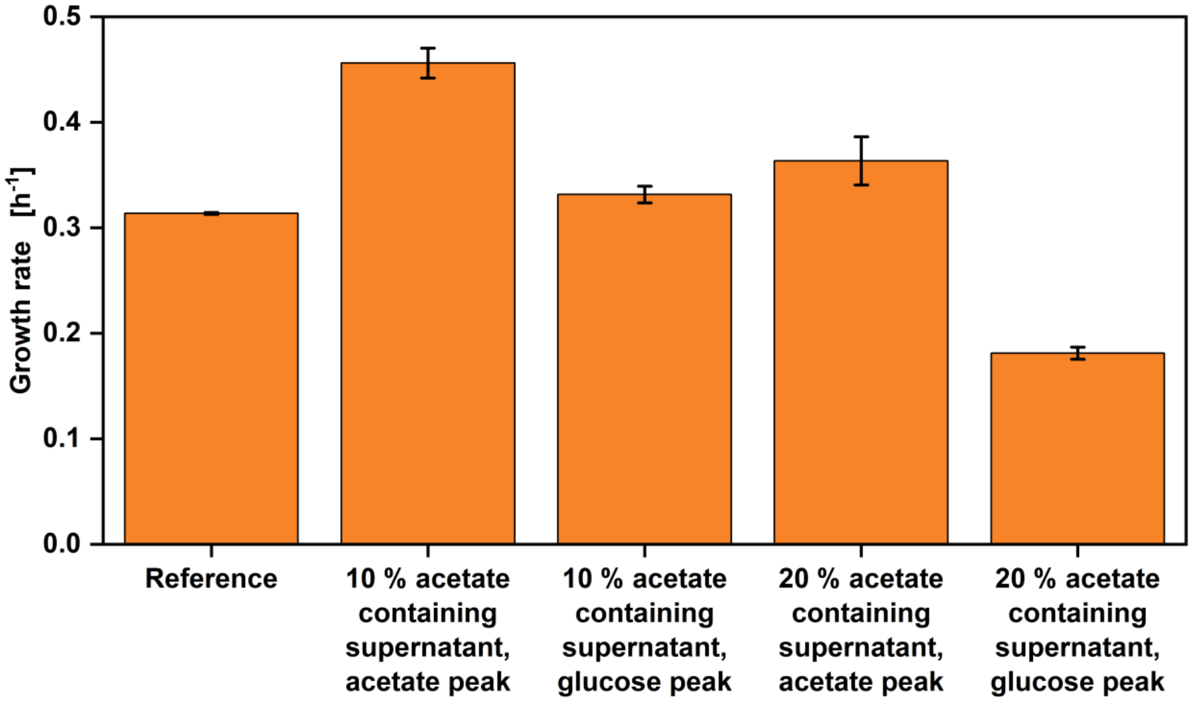

Figure S 2: **Growth rates for cultivation with *Corynebacterium glutamicum* DM 1933**. Reference, 10 % acetate containing supernatant (acetate spike), 10 % acetate containing supernatant (glucose peak), 20 % acetate containing supernatant (acetate peak), and 20 % acetate containing supernatant (glucose peak)

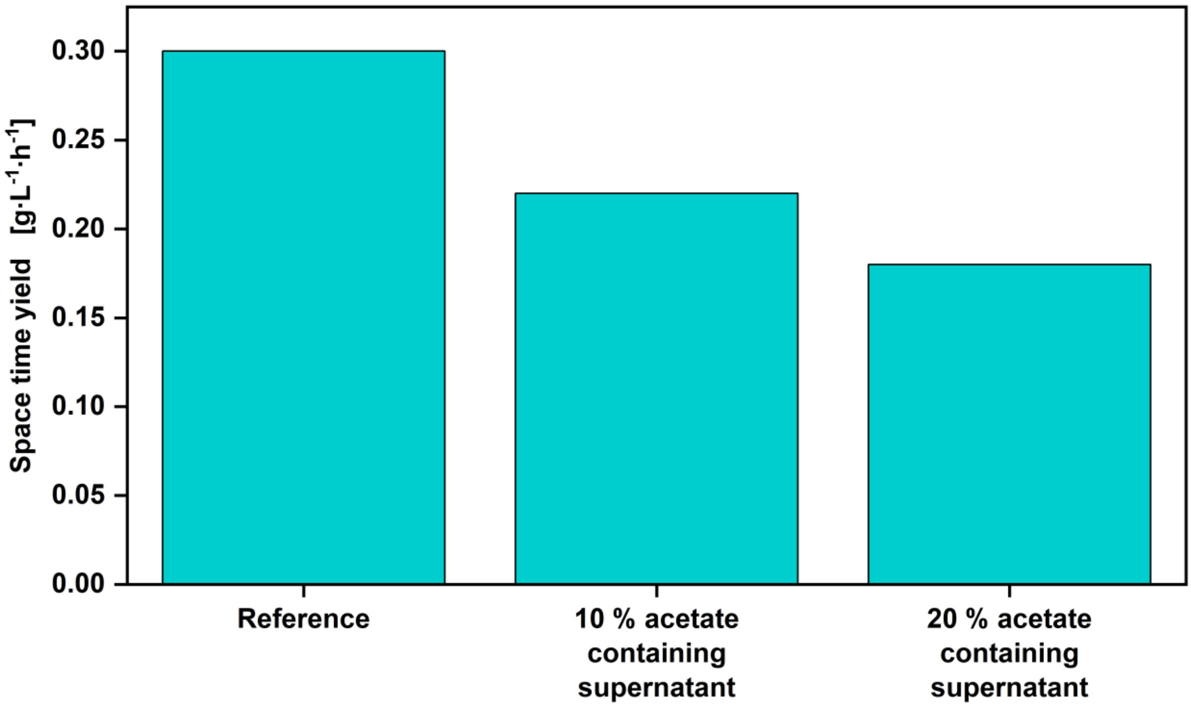

Figure S 3: Space-time yields of cultivations with *Corynebacterium glutamicum* DM 1933. Reference, 10 % acetate containing supernatant and 20 % acetate containing supernatant

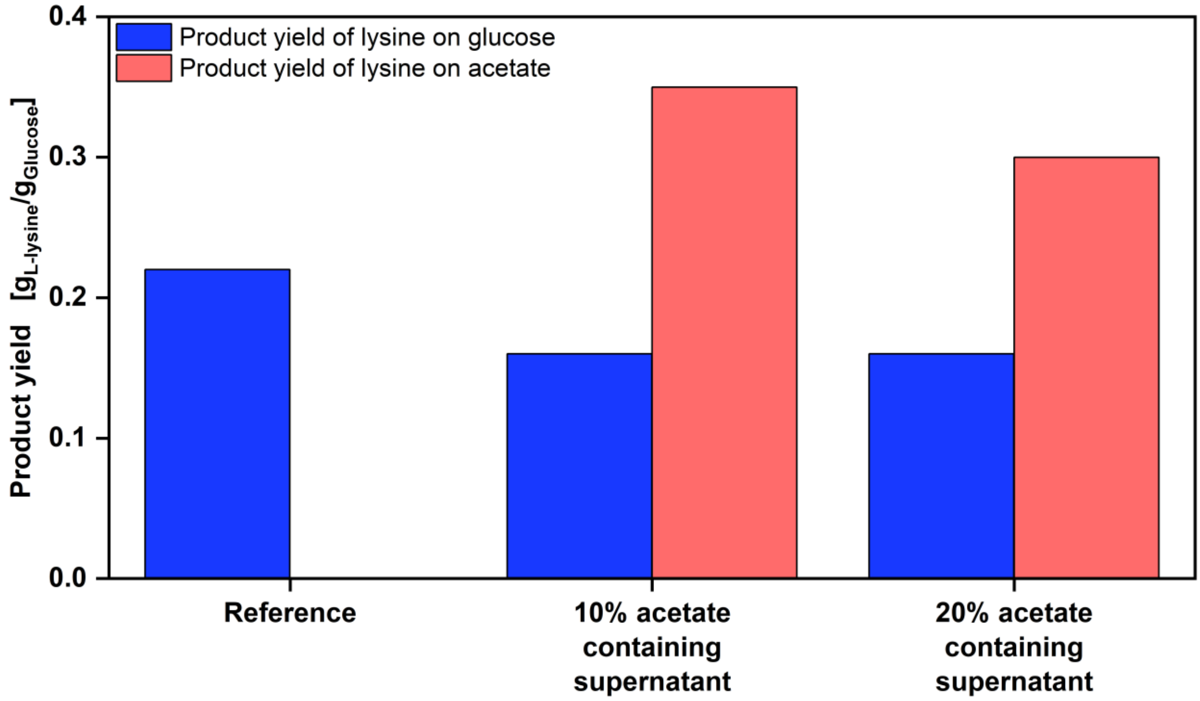

Figure S 4: Product yields of L-lysine depending on the metabolized carbon source during cultivation with *Corynebacterium glutamicum* DM 1933.

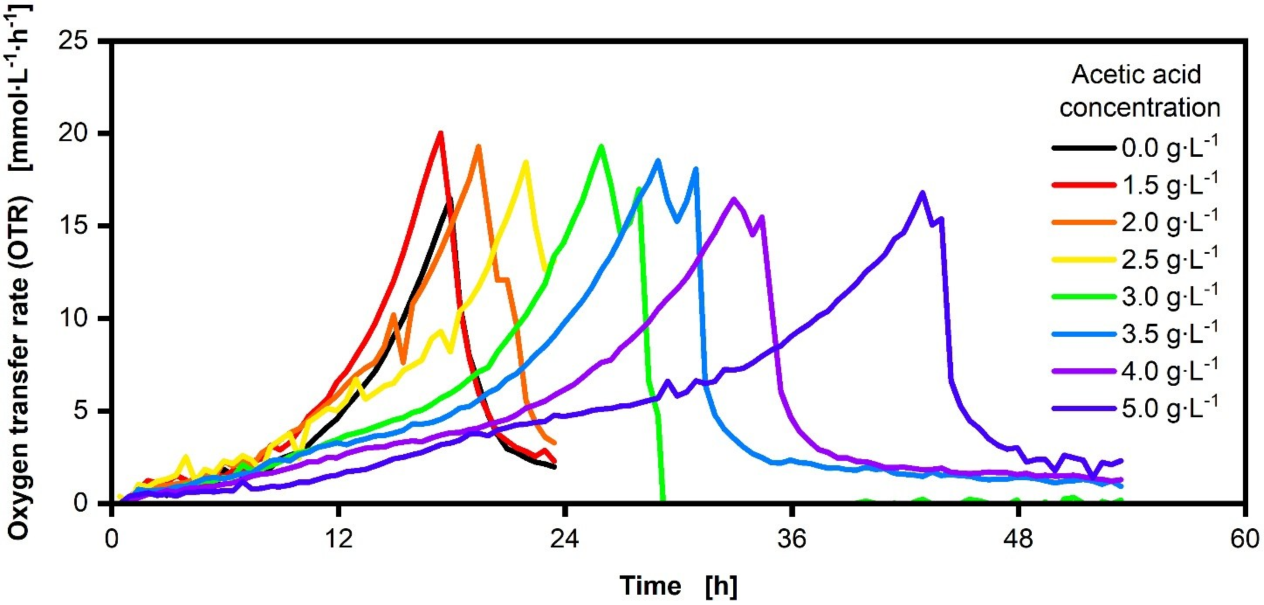

Figure S 5: Cultivation comparison of *U. maydis* MB215Δcyp1Δemt1 reference (black), with different acetic acid concentrations in modified Verduyn medium. The pH value of the complete media preparation, after the addition of acetic acid, was adjusted to 6.5 using 5 M NAOH. Cultivation conditions: 250 mL RAMOS shake flask, VL = 20 mL, d0 = 50 mm, n = 350 rpm, T = 30 °C, ODStart = 0.1 [-].

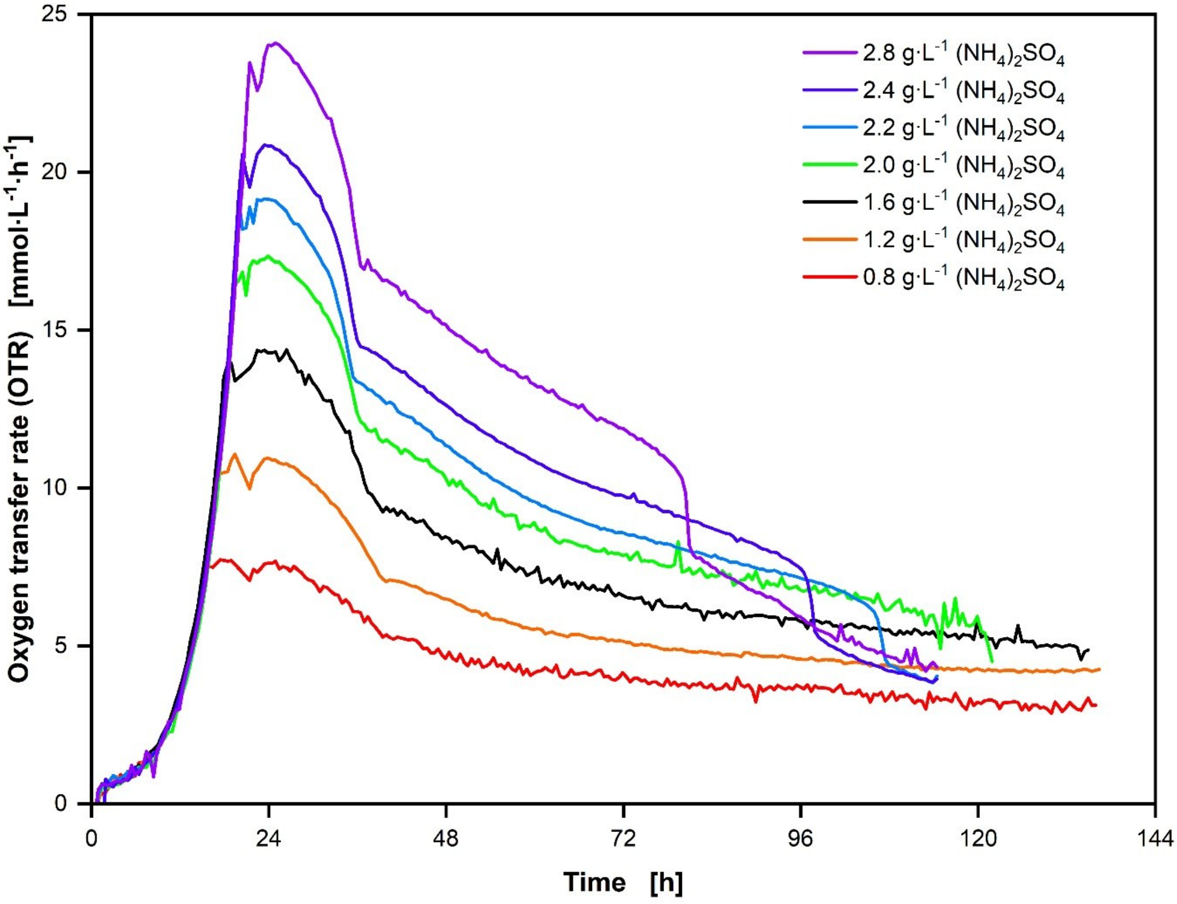

Figure S 6: Cultivation of *U. maydis* MB215Δcyp1Δemt1 with different nitrogen concentrations in modified Verduyn medium. Cultivation conditions: 250 mL RAMOS shake flask, V_L = 20 mL, d_0 = 50 mm, n = 350 rpm, T = 30 °C, OD_{Start} = 0.1 [-] c_{Glc} = 100 g·L^{-1}.

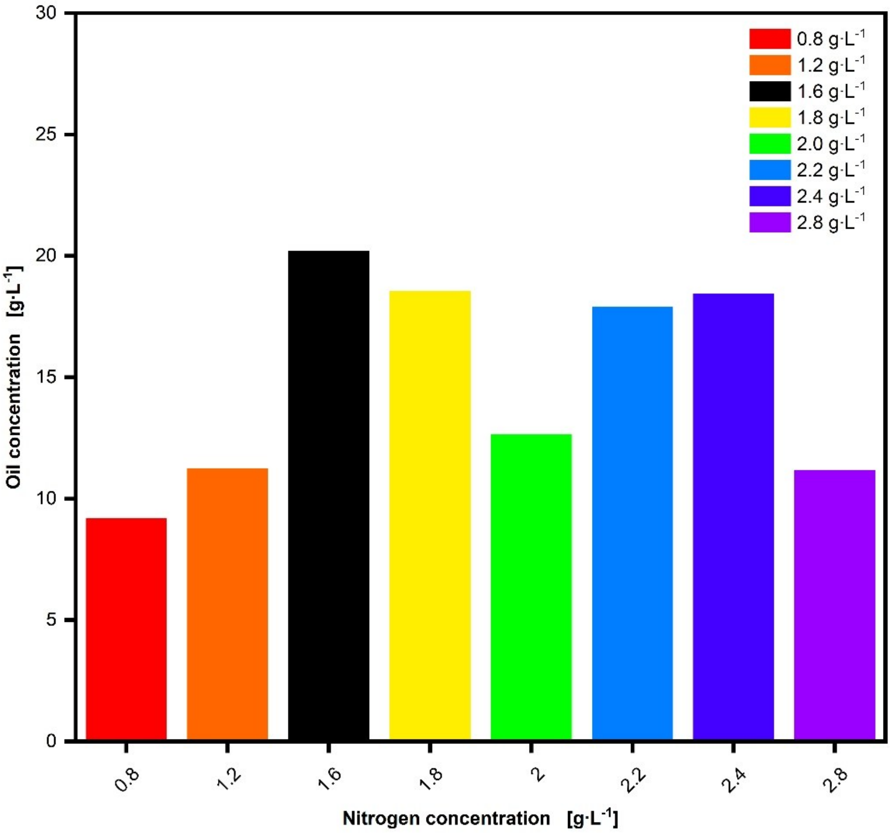

Figure S 7: Triglyceride concentration of different cultivations of *U. maydis* MB215Δcyp1Δemt1 with different nitrogen concentrations in modified Verduyn medium. Cultivation conditions: 250 mL RAMOS shake flask, V_L = 20 mL, d_0 = 50 mm, n = 350 rpm, T = 30 °C, OD_{Start} = 0.1 [-] c_{Glc} = 100 $g·L^{-1}$.
